# Conservative versus liberal oxygenation targets for mechanically ventilated patients: pilot multicentre randomised trial

**DOI:** 10.1186/2197-425X-3-S1-A423

**Published:** 2015-10-01

**Authors:** L Barrot, R Panwar, M Hardie, R Bellomo, G Eastwood, P Young, P Harrigan, M Bailey, G Capellier, CLOSE Study Investigators and the ANZICS Clinical Trials Group

**Affiliations:** CHRU Besançon, Medical ICU, Besancon, France; John Hunter Hospital, Newcastle, Australia; John Hunter Hospital, ICU, Newcastle, Australia; Department of Intensive Care, Austin Hospital, Melbourne, Australia; Austin Hospital, Intensive Care Unit, Melbourne, Australia; Wellington Hospital, Wellington, and Medical Research Institute of New Zealand, Intensive Care Unit, Wellington, New Zealand; John Hunter Hospital, Intensive Care Unit, Newcastle, Australia; Department of Epidemiology and Preventive Medicine, Australian and New Zealand Intensive Care Research Centre, Melbourne, Australia; CHRU Besançon, Intensive Care Unit, Besançon, France; Franche-Comté University, EA 3920 Besançon, France

## Introduction

There is now increasing recognition of potential harm due to hyperoxia. Conventional practice of oxygen therapy often result in hyperoxia. Nearly all patients on mechanical ventilation receive supplemental oxygen therapy. However, RCTs investigating the effects of different oxygenation level during MV are lacking

## Objectives

We aim to systematically evaluate whether liberal oxygenation strategy is beneficial or harmful compared to conservative oxygenation strategy for patients on invasive MV.

## Methods

Patients likely to require 48 hours of MV, older than 18 years were included. Prospective trial conducted in 4 university affiliated ICU in Australia, New Zealand and France. Enrolled patients were randomly allocated to either liberal or conservative oxygenation arm(SaO2/SpO2 >96% or between 88-92% respectively) and followed until day 90. The bedside nurse titrated FiO2 to reach the oxygenation target.

## Results

357 patients were screened and 104 were enrolled, 53 and 51 respectively in conservative and liberal arm respectively. Patients characteristics were similar at baseline. ABG’s were checked more often in the conservative arm. Using an hypoxemia threshold of SpO2 < 88%, 1% and 0.3% of the values in the conservative and liberal arm respectively were in hypoxemic range. With a PaO2 threshold < 55 mmHg, 7% and 1% respectively of the values were in hypoxemic range. With a threshold of SpO2>98% while FiO2 > 0.21, 4% and 22% of SpO2 values respectively were in the hyperoxic range. Same difference were found when using an hyperoxic threshold of PaO2>120 mmHg (p < 0.05 for all comparison). Participants spent majority of time within the intended target range in both groups. Mean AUC and 95%CI for SpO2, SaO2, PaO2 and FiO2 were significantly lower in the conservative group compared to liberal group. Survival analysis curve were similar for both groups (OR 0.77 (95%CI:0.4-1.50; p = 0.44)). For patients with a P/F ratio < 300, the separation in mean FiO2 was wider between the two arms. Outcome were similar. There slightly more arrhythmia-free days in the conservative arm. In thisP/F < 300 subgroup, the adjusted hazard ratio for death by day 90 was lower in the conservative arm compared to liberal arm.

## Conclusions

Our study demonstrate that conservative oxygenation strategy is a feasible and safe alternative to the usual liberal oxygenation strategy.

## Grant Acknowledgment

Intensive Care Foundation (ANZ), University Hospital Besançon (France) and Don Du Souffle (France).
